# Role of LATS1/2 in Prognosis of Advanced Gastric Cancer and Its Relationship With the Tumor Immune Microenvironment

**DOI:** 10.3389/fonc.2020.01406

**Published:** 2020-08-25

**Authors:** Yixian Guo, Xu Liu, Danhua Xu, Chen Huang, Zeyu Wang, Xiang Xia, Chunchao Zhu, Jia Xu, Zizhen Zhang, Yanying Shen, Wenyi Zhao, Gang Zhao

**Affiliations:** ^1^Department of Gastrointestinal Surgery, Renji Hospital, School of Medicine, Shanghai Jiao Tong University, Shanghai, China; ^2^Department of Pathology, Renji Hospital, School of Medicine, Shanghai Jiao Tong University, Shanghai, China

**Keywords:** gastric cancer, prognosis, LATS1/2, CD8, FOXP3, CD163, microsatellite stability

## Abstract

**Background:** Gastric cancer (GC) remains a refractory cancer particularly in Eastern Asia. Large tumor suppressor kinases 1/2 (LATS1/2) are core members of the Hippo pathway. The role of LATS1/2 in the prognosis of different subtypes of advanced gastric cancer and its relationship with the tumor immune microenvironment in GC remain unknown. Exploring the role of LATS1/2 in GC might provide potential immunotherapeutic approaches for treating GC.

**Methods:** Four hundred and ninety surgically resected primary GC samples were assessed for LATS1/2, CD8, FOXP3, and CD163. Correlations between LATS1/2 expression and immune-related markers were investigated and the prognoses of patients with different GC subtypes were analyzed.

**Results:** CD8 and CD163 appeared to be favorable and adverse prognostic factors, respectively. LATS1/2 and FOXP3 did not predict patients' overall survival. However, in microsatellite-stable GC patients, high LATS1/2 and FOXP3 expression and low CD8 expression predicted poor prognoses. Furthermore, high LATS1/2 expression was significantly correlated with decreased CD8 and increased FOXP3. Combined analysis of LATS1/2, CD8, and FOXP3 had better prognostic accuracy than did each marker individually.

**Conclusions:** Different biological molecules can predict the prognoses of different types of GC patients. LATS1/2, core kinases in the Hippo pathway, are closely related to CD8 and FOXP3. Further understanding the mechanisms of LATS1/2 in CD8^+^ T cells and FOXP3^+^ Treg cells provides further theoretical basis and potential targets for GC immunotherapy.

## Introduction

Gastric cancer (GC) is a serious malignant tumor with the fifth highest global incidence rate and the third highest mortality rate (1). Asian countries have high incidences of GC. The 5–year survival rate of GC patients in China is only 35.9% (2). GC patients are often diagnosed at advanced stages because of the lack of early characteristic symptoms and frequent recurrence and distant metastasis that occurs after surgical resection (3, 4). According to the Cancer Genome Atlas database, Bass et al. established a new molecular classification of GC (5). Cristescu et al. found that the prognoses of microsatellite-stable (MSS) and microsatellite-instable (MSI) GC patients differed in which patients with MSS GC had worse prognoses (6); however, the reason for this remains unclear. Therefore, using biological markers to analyze the prognoses of patients with different GC subtypes may provide clues for exploring the pathogenesis and clinical treatment of GC.

The Hippo pathway is a tumor-suppressive pathway, and its inactivation is associated with the progression and metastasis of various cancers (7, 8). Large tumor suppressor kinases 1/2 (LATS1/2) are core members of the Hippo pathway, and their activation is the major functional output of this pathway. LATS1 and LATS2 have the same function and are both expressed in GC (9, 10). Although LATS1/2 are traditionally believed to inhibit tumor growth (11, 12), Pan et al. found that LATS1/2 deletion inhibits the growth of murine MC38 colon cancer cells (13). Moreover, LATS1/2 inhibit antitumor immunity by suppressing CD8 cytotoxicity. Mechanistically, LATS1/2-null tumor cells secrete nucleic acid-rich extracellular vesicles, which induce a type I interferon response via the Toll-like receptor-MYD88/TRIF pathway (14). Therefore, the role of LATS1/2 in a tumor microenvironment remains controversial. To explore the relationship between LATS1/2 and a tumor microenvironment in advanced GC, we used tumor immune-related markers including CD8, FOXP3, and CD163, representing CD8^+^ T cells, FOXP3^+^ Treg cells, and CD163^+^ M2 macrophages, respectively, and all played important roles in a tumor immune microenvironment (15–20). We sought to identify novel strategies to obtain more accurate prognoses in advanced GC patients by analyzing different biological marker combinations.

We found that different biological markers predicted the prognoses of patients with different types of advanced GC. LATS1/2, important kinases in the Hippo pathway, were closely related to CD8 and FOXP3. Furthermore, we identified novel strategies for obtaining more accurate prognoses in GC patients by analyzing LATS1/2 in combination with immune-related markers including CD8 and FOXP3.

## Materials and Methods

### Patients

This study was conducted on a cohort of 490 patients with advanced GC [American Joint Committee on Cancer (AJCC) stages T2–T4]. All samples were retrieved from patients who underwent primary tumor resection between June 2006 and December 2016 at the Department of Gastrointestinal Surgery, Renji Hospital, School of Medicine, Shanghai Jiao Tong University. All samples were definitively diagnosed as advanced GC by the Department of Pathology. The clinical criteria for patient recruitment were as follows: (i) patients had complete clinical information, postoperative pathological diagnoses, and follow-up data; (ii) patients had not received preoperative radiotherapy, chemotherapy, hormonal therapy, or any other anticancer therapy before surgery; (iii) patients had undergone non-neoplastic resection such as laparotomy or palliative gastrointestinal bypass surgery; (iv) patients had non-adenocarcinoma (gastrointestinal stromal tumors); and (v) the primary tumor involved only one regional site at the site of occurrence (21, 22).

Overall survival time was defined as the interval between gastrectomy and either patient death or the last follow-up. The final follow–up date was February 25, 2020. All patients received standard treatments such as D2 radical resection and adjuvant chemotherapy or palliative tumor resection for patients with stage IV GC according to the National Comprehensive Cancer Network (NCCN) guidelines. Patients' tumors were staged in accordance with the AJCC 8th edition staging system. Two senior pathologists confirmed the diagnosis in each case from the hematoxylin and eosin-stained slides.

### Immunohistochemistry

Formalin-fixed, paraffin-embedded (FFPE) tissue samples were sliced in consecutive 3.0-μm-thick sections, which were dewaxed in xylene and rehydrated in graded ethanol. Immunohistochemical staining was then performed as per the Dako REAL EnVision Detection System (K5007, Dako) manual. The following primary antibodies were used:

Anti-LATS1/2 (1:100; ab111344, Abcam), Anti-CD163 (1:100, ab87099, Abcam), Anti-CD8 (1:100, ab4055, Abcam), Anti-FOXP3 (1:100, ab20034, Abcam), Anti-MLH1 (1:50, clone ES05, DAKO), Anti-PMS2 (1:40, clone EP51, DAKO), Anti-MSH2 (1:50, clone FE11, DAKO), and Anti-MSH6 (1:50, clone EP49, DAKO).

Paraffin-embedded sections (3.0 μm) were prepared for immunohistochemical analyses. After deparaffinization, all antigens except nestin were retrieved at 120°C for 15 min in a sodium citrate buffer solution (pH 6.0). Tissues were incubated with 0.3% hydrogen peroxide for 30 min and then blocked with 1% bovine serum albumin (Sangon, Shanghai, China) overnight at 4°C. The peroxidase reaction was developed using a 3,3-diaminobenzidine (DAB) chromogen solution in a DAB buffer substrate and then counterstained with hematoxylin.

### Histological Scoring

Five random fields per section were viewed under a light microscope (Axioskop 40; Zeiss GmbH, Jena, Germany) at 400× magnification. Three investigators, who were blinded to the clinical features and outcomes, independently examined and scored the sections. After counting the cells, the cell density was calculated as mm^2^ for further analysis.

In a two-category immunoscore analysis, patients were dichotomized into the high- and low-density groups according to the median number of stained cells. The cutoffs were as follows: 17/mm^2^ for CD8, 25/mm^2^ for FOXP3, and 20/mm^2^ for CD163 (23, 24). LATS1/2 expressions in the samples were considered high when they were expressed in at least 10% of the samples (25).

### Statistical Analyses

All statistical analyses were performed using SPSS 23.0 and GraphPad Prism 8.0. The Spearman's correlation coefficient was calculated to examine associations between continuous variables. Chi-square tests were performed to analyze relationships between categorical variables. Kaplan–Meier univariate and multivariate prognostic analyses of the Cox proportional hazards regression model were performed to assess the influence of each variable on survival. Hazard ratios (HRs) with 95% confidence intervals (CIs) were calculated as correlation estimates. A two-tailed *P* < 0.05 was considered statistically significant.

## Results

### Patients' Clinicopathologic Characteristics

Four hundred ninety surgically resected FFPE primary advanced GC samples were assessed for LATS1/2, CD8, FOXP3, and CD163 via tissue microarrays. The patients included 337 men (68.78%) and 153 women (31.22%). The median age at diagnosis was 62 years old (range: 22–88 years). One hundred seventy-two patients (35.10%) were <60 years old, and 318 (64.90%) were >60 years old. The median overall survival time was 43 months (range: 0–123 months). One hundred seventeen GC lesions (23.88%) occurred in the upper stomach, 157 (32.04%) occurred in the middle stomach, and 216 (44.08%) occurred in the lower stomach. The tumors were classified based on the 8th AJCC gastric cancer staging manual: 150 were stage II (30.61%), 266 were stage III (54.29%), and 74 were stage IV (15.10%) (26). According to the 8th AJCC staging, lymph node metastasis occurred in 366 cases (74.69%), while distant metastasis occurred in 74 cases (15.10%). The tissue samples comprised 200 cases (40.82%) of intestinal type carcinoma and 290 cases (59.18%) of diffused gastric carcinoma. According to the microsatellite stability classification, 364 cases were MSI (74.29%) and 126 were MSS (25.71%; Table 1).

**Table 1:** Characteristics of 490 advanced gastric cancer (GC) patients.

**Patient characteristics**	**Number**	**% of total**
All patients	490	
**Sex**		
Male	337	68.78%
Female	153	31.22%
**Age**		
≤ 60 years	172	35.10%
>60 years	318	64.90%
**Location**		
Upper	117	23.88%
Middle	157	32.04%
Low	216	44.08%
**AJCC TNM stage**		
Stage II	150	30.61%
Stage III	266	54.29%
Stage IV	74	15.10%
All patients	490	
**T-stage**		
T1	2	0.41%
T2	42	8.57%
T3	141	28.78%
T4	305	62.24%
**Lymph node metastasis**		
N0	124	25.31%
N1	366	74.69%
**Distant metastasis**		
M0	416	84.90%
M1	74	15.10%
**Lauren classification**		
Intestinal	200	40.82%
Diffused	290	59.18%
**Microsatellite stability**		
MSS	364	74.29%
MSI	126	25.71%

*AJCC, American Joint Committee on Cancer; N0, no lymph node metastasis; N1, lymph node metastasis; M0, no distant metastasis; M1, distant metastasis; MSS, microsatellite stability; MSI, microsatellite instability*.

### Association Between LATS1/2, CD8, FOXP3, CD163, and Clinical Characteristics

We classified biomarkers according to the expression level via microscopic observation. Figures S1, S2 show the LATS1/2, CD8, FOXP3, and CD163 expression profiles. LATS1/2 were highly expressed in 226 cases (46.12%) and lowly expressed in 264 cases (53.88%). CD8 was highly expressed in 245 cases (50%) and lowly expressed in 245 cases (50%). CD163 was lowly expressed in 257 cases (52.44%) and highly expressed in 233 cases (47.55%). FOXP3 was highly expressed in 213 cases (43.46%) and lowly expressed in 277 cases (56.54%; Table S1).

Table S1 shows the association between LATS1/2, CD8, FOXP3, CD163, and the pathological features. LATS1/2 were significantly positively correlated with AJCC stage (*P* = 0.049) and microsatellite stability (*P* = 0.041). CD8 expression was significantly negatively associated with the AJCC stage (*P* = 0.039), the advanced tumor stage (*P* = 0.035), distant metastasis (P = 0.023), the Lauren classification (*P* = 0.043), and microsatellite stability (*P* = 0.032). FOXP3 was significantly correlated with microsatellite stability (*P* = 0.042). CD163 was not correlated with any of the pathological features.

### Survival Analysis of Clinicopathological Features and LATS1/2, CD8, FOXP3, and CD163 Expressions in Advanced GC

We analyzed the relationship between prognosis and clinical features in patients with GC via Cox regression analysis. The tumor node metastasis (TNM) stage (HR = 1.353, 95% CI: 1.116–1.640, *P* = 0.002), lymph node metastasis (HR = 1.827, 95% CI: 1.363–2.451, *P* = 0.000), distance metastasis (HR = 3.377, 95% CI: 2.546–4.480, *P* = 0.000), Lauren classification (HR = 1.530, 95% CI: 1.324–1.885, *P* = 0.000), and microsatellite classification (HR = 1.336, 95% CI: 1.083–1.729, *P* = 0.009) were significantly associated with the overall survival (Table S2). Cox regression analysis was performed to evaluate the prognostic roles of LATS1/2, CD8, FOXP3, and CD163. Univariate analysis showed that CD8 (HR = 0.706, 95% CI: 0.559–0.893, *P* = 0.004) and CD163 (HR = 1.222, 95% CI: 1.089–1.417, *P* = 0.033) predicted patients' prognoses. However, LATS1/2 (HR = 1.157, 95% CI: 0.917–1.461, *P* = 0.219) and FOXP3 (HR = 1.110, 95% CI: 0.878–1.402, *P* = 0.384) expressions did not significantly affect the overall survival.

**Figure 1:**
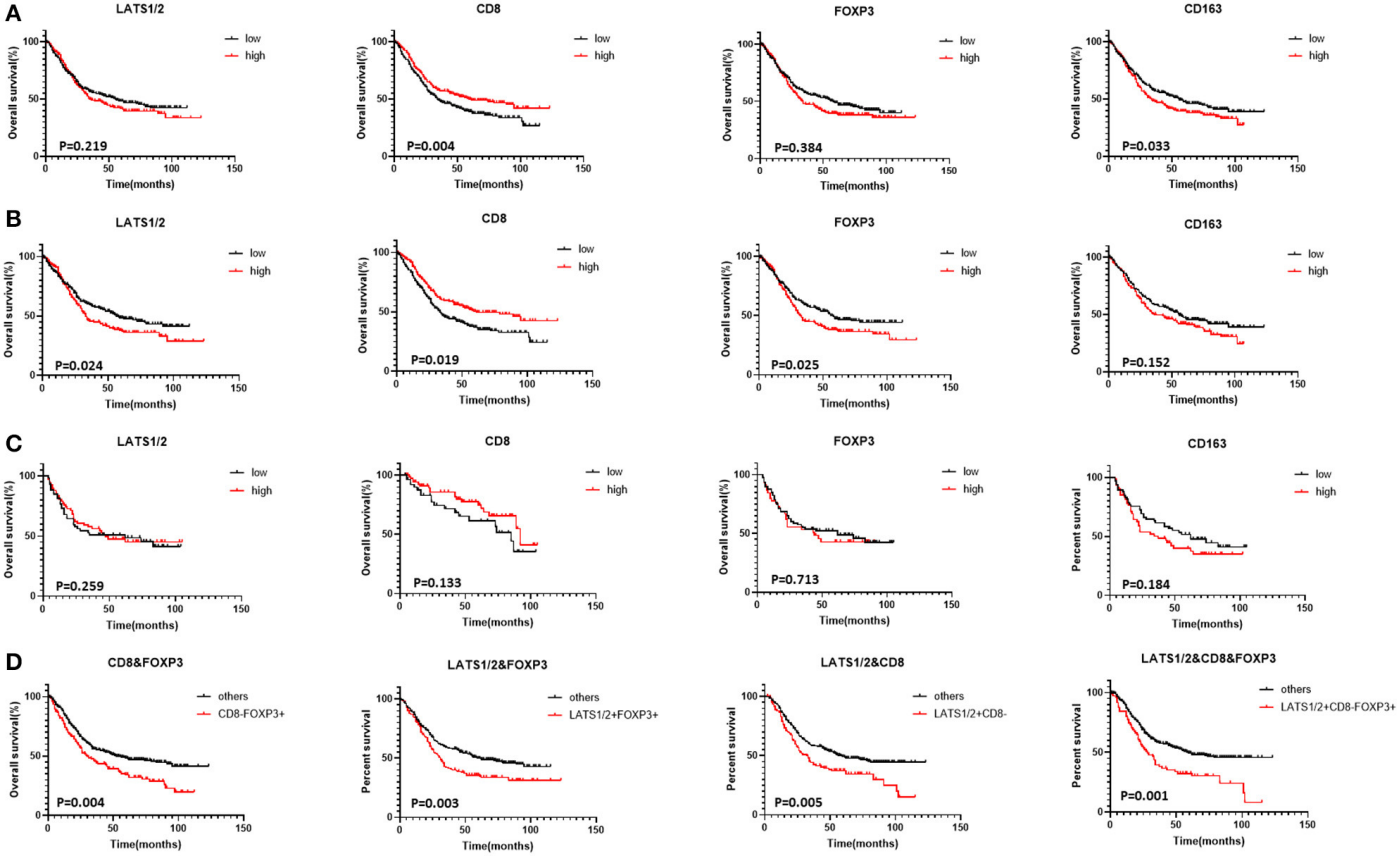
Correlation between LATS1/2, CD8, FOXP3, and CD163 and GC patients' overall survival (Kaplan–Meier survival curves): **(A)** patients with advanced GC, **(B)** patients with advanced MSS GC, **(C)** patients with advanced MSI GC, and **(D)** combination of LATS1/2, CD8, and FOXP3 in all patients with advanced GC.

Variables with *P* < 0.05 in the univariate analysis were included in multivariate analyses. Because TNM staging included both lymph node metastasis and distant metastasis, they were excluded. The TNM stage (HR = 1.316, 95% CI: 1.087–1.599, *P* = 0.046) and CD8 (HR = 0.705, 95% CI: 0.556–0.893, *P* = 0.004) were independent factors for predicting the overall survival in the multivariate analysis. LATS1/2 and FOXP3 did not predict patient prognoses (Figure 1A, Table S2).

Patients were then divided into MSS and MSI subgroups according to MLH1, MSH2, MSH6, and PMS2 expressions (27, 28). FOXP3 and CD163 expressions were significantly higher in MSS GC patients than in MSI GC patients while CD8 expression was significantly lower in MSS GC patients (Table S1). Although LATS1/2 and FOXP3 expressions did not predict advanced GC, high LATS1/2 (HR = 1.304, 95% CI: 1.035–1.643, *P* = 0.024) and FOXP3 (HR = 1.320, 95% CI: 1.047–1.665, *P* = 0.019) expressions predicted shorter overall survival in patients with MSS GC. In patients with MSI GC, LATS1/2, CD8, FOX3, and CD163 expressions did not significantly affect the overall survival (Figures 1B,C, Table S3).

### Prognostic Value of the Combination of LATS1/2, FOXP3, and CD163 in Advanced GC

To evaluate LATS1/2 expression in cells in the immune microenvironment and the relationship between LATS1/2 and immune cells, we first analyzed the relationship between LATS1/2, FOXP3, CD163, and CD8 (Table S4). High LATS1/2 expression was significantly correlated with low CD8 expression (*P* = 0.008) and high FOXP3 expression (*P* = 0.012), but LATS1/2 and CD163 were not correlated. Thus, we combined LATS1/2 with CD8 and FOXP3 for prognostic analysis and divided them into four subtypes: subtype 1 with LATS1/2 and CD8; subtype 2 with LATS1/2 and FOXP3; subtype 3 with CD8 and FOXP3; and subtype 4 with LATS1/2, CD8, and FOXP3. The survival curve revealed that the LATS1/2^high^CD8^low^, LATS1/2^high^FOXP3^high^, CD8^low^FOXP3^high^, and LATS1/2^high^CD8^low^FOXP3^high^ subgroups in each subtype have the worst overall survival (Figures S3A–D). We then compared them with other subgroups (Figure 1D, Table S5). Combined analysis of the three indicators, LATS1/2, CD8, and FOXP3, had better prognostic accuracy than did the combination of any two indicators (HR = 2.207, 95% CI: 1.653–2.959, *P* = 0.001). Thus, the combined analysis of LATS1/2, CD8, and FOXP3 may be a good prognostic factor for patients with advanced GC.

## Discussions

LATS1/2 are key kinases in the Hippo signaling pathway. LATS1/2 activation can inhibit tumor growth (29, 30); however, Toshiro et al. recently reported that suppressing LATS1/2 exhibited antitumor immunity (14); therefore, the roles of LATS1/2 in the tumor microenvironment remain controversial. Here, we selected tumor immunity-related biological markers, including CD8, FOXP3, and CD163, which, respectively, represented CD8^+^ T cells, FOXP3^+^ Treg cells, and CD163^+^ M2 macrophages to analyze different advanced GC types. We focused on the relationship between LATS1/2 and these tumor immunity-related biological markers in a tumor immune microenvironment by analyzing 490 immunohistochemically stained samples from advanced GC patients.

First, we analyzed the correlation between LATS1/2, CD8, FOXP3, CD163, and clinicopathological features and prognosis of patients with advanced GC. CD8 and CD163 represented favorable and adverse prognostic factors, respectively. High LATS1/2 expression in GC has been reported to yield better prognoses (9). However, LATS1/2 and FOXP3 expressions did not predict the overall survival in patients with advanced GC in this study. The results revealed that high LATS1/2 expression was related to TNM stage progression. Different GC types may have different prognosis-related biological indicators (24). Therefore, LATS1/2 may differently predict the prognosis in different GC subtypes. Because LATS1/2 is significantly related to GC microsatellite stability, we then analyzed LATS1/2 expressions in MSS and MSI patients.

Molecular classification is essential for subtyping GC (5, 6). The prognoses differ among patients with different molecular subtypes, but the reason remains unclear (6). Therefore, we defined patients with MSS GC and MSI GC according to the immunohistochemical expressions of MLH1, MSH2, MSH6, and PMS2 (28, 29). Patients with MSS GC had poor prognoses, which is consistent with previous reports (29). In addition, LATS1/2 and FOXP3 expressions were increased while CD8 expression was decreased in patients with MSS GC. CD163 expression did not significantly differ between MSS GC and MSI GC patients. Next, we separately analyzed the prognoses of MSS and MSI GC patients according to LATS1/2, CD8, FOXP3, and CD163 expressions. High CD163 expression indicated a poor prognosis for GC patients. Subgroup analysis revealed that CD163 did not predict the prognoses of MSS and MSI patients, but the results revealed that its survival prognosis trend was consistent with that of the overall analysis and is likely because the decreased sample sizes resulted in no statistical differences after grouping. Therefore, in future studies, we should further analyze the role of CD163 in different GC subgroups by increasing the sample size and continuing to follow the patients. Furthermore, in MSS GC patients, high LATS1/2 and FOXP3 expressions and low CD8 expression predicted adverse patient prognoses, whereas in MSI GC, only CD8 predicted patient prognoses. In patients with MSS GC, LATS1/2 signaling pathway activation was also correlated with an adverse prognosis, indicating that LATS1/2 activation might suppress the antitumor effect of CD8^+^ T cells and activate immunosuppressive effects in FOXP3^+^ Treg cells. However, its deep mechanism remains unclear. Ma et al. reported that PD-L1 was always expressed in MSI and EBV(+) GC. Kim et al. found that anti-PD1 treatment was more effective against MSI and EBV(+) GC (31, 32), but less attention was paid to MSS GC. Of note, we found that LATS1/2 were more highly expressed in MSS GC than in MSI GC and could be regarded as adverse prognostic factors in MSS GC, suggesting that LATS1/2 might be a new target for MSS GC treatment.

Recent evidence revealed that combined analysis of multiple biological markers has a superior prognostic value compared with that of analyzing individual biological markers (23). We found that LATS1/2 were negatively correlated with CD8 and positively correlated with FOXP3. We combined LATS1/2, CD8, and FOXP3 to analyze the prognoses of advanced GC patients and found that combined analysis of LATS1/2, CD8, and FOXP3 predicted patient prognoses, and the HR of the combined analysis of the three indicators was better than the combination of any two indicators, suggesting that LATS1/2 might play an important role in CD8^+^ T cells and FOXP3^+^ Treg cells in a tumor immune microenvironment. CD8^+^ T cells and FOXP3^+^ Treg cells are reported to be closely related and play important roles in tumor development and immune escape in breast, ovarian, and gastric cancers (33–35). In a tumor immune microenvironment, LATS1/2 knockout in tumor cells weakened the CD8^+^T cell functions, leading to tumor immune escape (13). In GC, a previous report indicated that LATS2 was positively correlated with FOXP3 (36), but the function of LATS1/2 in FOXP3^+^ Treg cells is unreported and may represent a future research direction. Thus, combined analysis of LATS1/2, CD8, and FOXP3 might be a good strategy for obtaining an accurate prognosis in advanced GC patients.

This study has several limitations. First, the data in our analysis was from a single center without an external validation cohort and needs to be jointly verified by multiple centers. Second, this was a retrospective study and thus was inherently subject to selection bias.

In summary, LATS1/2, CD8, and FOXP3 expressions may be used as prognostic markers in advanced GC patients. Our study identified the novel individual marker— LATS1/2, for obtaining a prognosis in MSS GC, and combining LATS1/2, CD8, and FOXP3 may serve as a prognostic marker in advanced GC. These findings contribute to better understand advanced GC, and further investigation is needed to elucidate the underlying mechanisms of these markers.

## Data Availability Statement

The raw data supporting the conclusions of this article will be made available by the authors, without undue reservation.

## Ethics Statement

The studies involving human participants were reviewed and approved by Ethical Committee of the Shanghai Jiao Tong University School of Medicine, Renji Hospital. The patients/participants provided their written informed consent to participate in this study. Written informed consent was obtained from the individual(s) for the publication of any potentially identifiable images or data included in this article.

## Author Contributions

XL and DX collected the data and wrote the manuscript. YG, CH, and ZW analyzed the data and contributed in writing the manuscript. GZ and WZ performed the design and oversaw the project. YS provided the immunohistochemical analysis. XX, CZ, ZZ, JX, and GZ substantially contributed to the study design, performed the surgery, and revised the manuscript. All authors contributed to the article and approved the submitted version.

## Conflict of Interest

The authors declare that the research was conducted in the absence of any commercial or financial relationships that could be construed as a potential conflict of interest.
