# Supplementary material for: Role of LATS1/2 in Prognosis of Advanced Gastric Cancer and Its Relationship With the Tumor Immune Microenvironment
Source: Front Oncol. 2020 Aug 25;10:1406. doi: 10.3389/fonc.2020.01406 (PMC7477306; doi:10.3389/fonc.2020.01406)
Supplement: Supplementary file 1 [file Data_Sheet_1.docx]

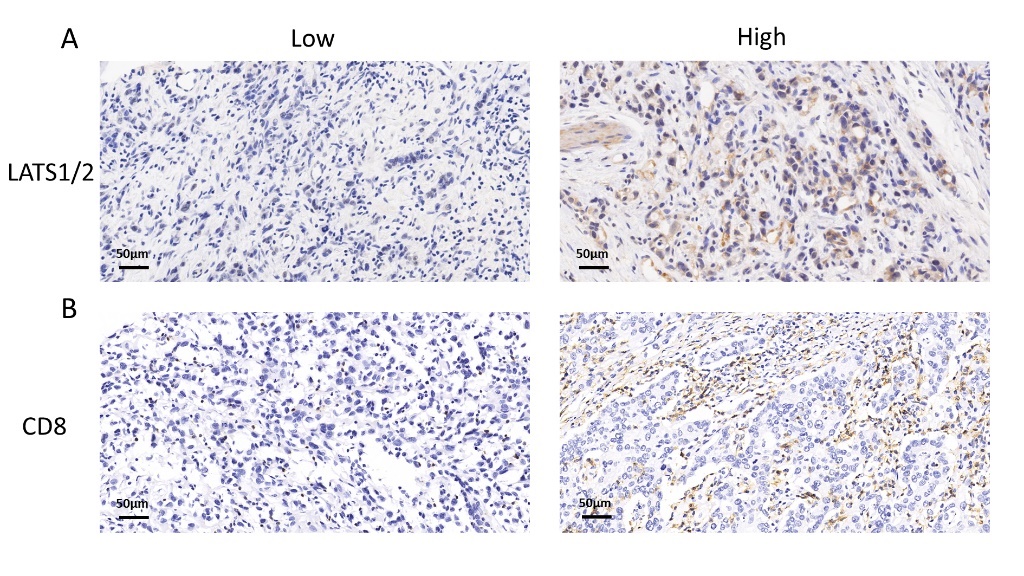


Figure S1. LATS1/2 and CD8 expression levels in advanced GC via immunohistochemistry

A. Low and high expression of LATS1/2. B. Low and high expression of CD8.


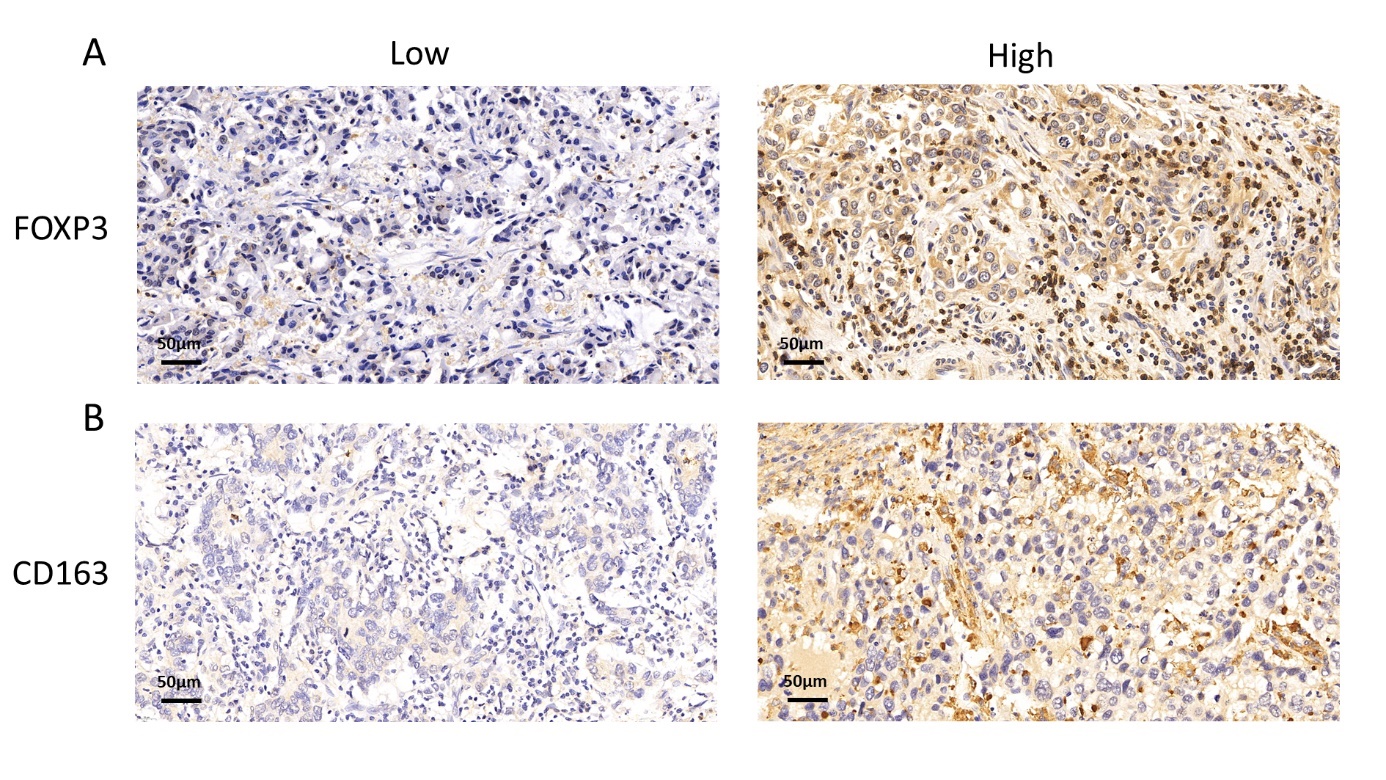


Figure S2. FOXP3 and CD163 expression levels in advanced GC via immunohistochemistry

A. Low and high expression of FOXP3. B. Low and high expression of CD163.


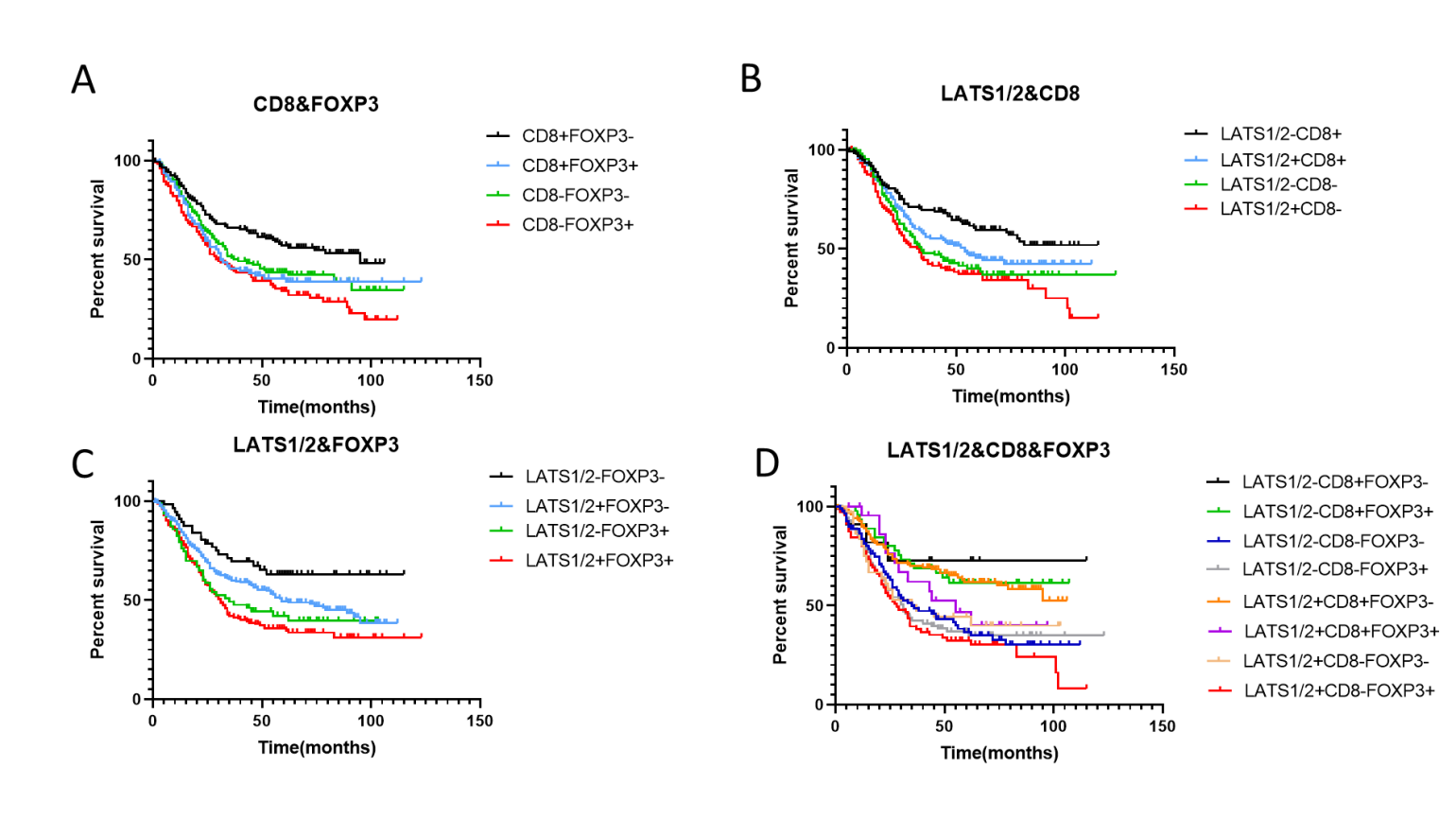


**Figure S3. Combined analysis of LATS1/2, CD8, FOXP3 and CD163 in survival of gastric cancer patients**

A. subtype 1, CD8+FOXP3; B. subtype 2, LATS1/2+CD8; C. subtype 3, LATS1/2+FOXP3; D. subtype 4, LATS1/2+CD8+FOXP3.

### Table S1. Clinicopathologic characteristics of 490 advanced gastric cancer patients

| Clinicopathological factors | CD8 | | | FOXP3 | | | CD163 | | | LATS1/2 | | |
| --- | --- | --- | --- | --- | --- | --- | --- | --- | --- | --- | --- | --- |
|  | low | high | P-value | low | high | P-value | low | high | P-value | low | high | P-value |
| Sex |  |  |  |  |  |  |  |  |  |  |  |  |
| Male | 165 | 172 | 0.496 | 193 | 144 | 0.611 | 168 | 169 | 0.141 | 182 | 155 | 0.553 |
| Female | 80 | 73 |  | 84 | 69 |  | 89 | 64 |  | 82 | 71 |  |
| Age |  |  |  |  |  |  |  |  |  |  |  |  |
| <60 years | 91 | 81 | 0.344 | 101 | 71 | 0.472 | 95 | 77 | 0.395 | 98 | 74 | 0.311 |
| ≥60 years | 154 | 164 |  | 176 | 142 |  | 162 | 156 |  | 166 | 152 |  |
| Location |  |  |  |  |  |  |  |  |  |  |  |  |
| Upper | 56 | 61 | 0.534 | 61 | 56 | 0.468 | 60 | 57 | 0.229 | 55 | 62 | 0.666 |
| Middle | 75 | 82 |  | 90 | 67 |  | 86 | 71 |  | 84 | 73 |  |
| Low | 114 | 102 |  | 126 | 90 |  | 111 | 105 |  | 125 | 91 |  |
| AJCC TNM-stage |  |  |  |  |  |  |  |  |  |  |  |  |
| pT2 | 61 | 89 | 0.039 | 91 | 59 | 0.163 | 90 | 60 | 0.057 | 92 | 58 | 0.049 |
| pT3 | 142 | 124 |  | 151 | 115 |  | 134 | 132 |  | 136 | 130 |  |
| pT4 | 42 | 32 |  | 35 | 39 |  | 33 | 41 |  | 34 | 40 |  |
| T-stage |  |  |  |  |  |  |  |  |  |  |  |  |
| T1 | 1 | 1 | 0.035 | 1 | 1 | 0.08 | 1 | 1 | 0.126 | 1 | 1 | 0.075 |
| T2 | 22 | 20 |  | 23 | 19 |  | 24 | 18 |  | 26 | 16 |  |
| T3 | 80 | 61 |  | 88 | 53 |  | 85 | 56 |  | 82 | 59 |  |
| T4 | 142 | 163 |  | 165 | 140 |  | 148 | 157 |  | 155 | 150 |  |
| Lymph node metastasis | |  |  |  |  |  |  |  |  |  |  |  |
| N0 | 71 | 53 | 0.087 | 77 | 47 | 0.239 | 60 | 64 | 0.367 | 74 | 50 | 0.212 |
| N1 | 174 | 192 |  | 200 | 166 |  | 197 | 169 |  | 190 | 176 |  |
| Distant metastasis |  |  |  |  |  |  |  |  |  |  |  |  |
| M0 | 203 | 213 | 0.023 | 242 | 174 | 0.098 | 224 | 192 | 0.090 | 230 | 186 | 0.100 |
| Ml | 42 | 32 |  | 35 | 39 |  | 33 | 41 |  | 34 | 40 |  |
| Lauren classification | |  |  |  |  |  |  |  |  |  |  |  |
| intestinal | 89 | 111 | 0.043 | 104 | 96 | 0.093 | 114 | 86 | 0.101 | 116 | 84 | 0.128 |
| diffuse | 156 | 134 |  | 173 | 117 |  | 143 | 147 |  | 148 | 142 |  |
| Microsatellite stability | |  |  |  |  |  |  |  |  |  |  |  |
| MSS | 192 | 172 | 0.032 | 196 | 168 | 0.042 | 191 | 173 | 0.412 | 186 | 178 | 0.041 |
| MSI | 53 | 73 |  | 81 | 45 |  | 66 | 60 |  | 78 | 48 |  |

AJCC: American Joint Committee on Cancer; N0: no lymph node metastasis; N1: lymph node metastasis; M0: no distant metastasis; M1: distant metastasis; MSI: microsatellite instability; MSS: microsatellite stability

### Table S2. [Univariable and multivariable analysis of 490 advanced gastric cancer](https://static-content.springer.com/esm/art%3A10.1186%2Fs12967-019-1929-9/MediaObjects/12967_2019_1929_MOESM2_ESM.docx) patients

| Clinicopathological factors | Univariate analysis | | | | Multivariate analysis | | | |
| --- | --- | --- | --- | --- | --- | --- | --- | --- |
|  | HR | 95%CI | | P-value | HR | 95%CI | | P-value |
| Age |  |  |  |  |  |  |  |  |
| <60 years | 1.010 | 0.998 | 1.022 | 0.094 |  |  |  |  |
| ≥60 years |  |  |  |  |  |  |  |  |
| Sex |  |  |  |  |  |  |  |  |
| Male | 0.958 | 0.746 | 1.231 | 0.738 |  |  |  |  |
| Female |  |  |  |  |  |  |  |  |
| AJCC TNM-stage | | | | | | | | |
| pT4  pT2-pT3 | 1.353 | 1.116 | 1.640 | 0.002 | 1.316 | 1.087 | 1.599 | 0.046 |
| Lymph node metastasis | | | | | | | | |
| N1 | 1.827 | 1.363 | 2.451 | 0.000 |  |  |  |  |
| N0 |  |  |  |  |  |  |  |  |
| Distant metastasis | | | | | | | | |
| M1 | 3.377 | 2.546 | 4.480 | 0.000 |  |  |  |  |
| M0 |  |  |  |  |  |  |  |  |
| Lauren classification | | | | | | | | |
| Diffuse | 1.530 | 1.324 | 1.885 | 0.000 | 1.056 | 0.781 | 1.428 | 0.722 |
| Intestinal |  |  |  |  |  |  |  |  |
| Microsatellite stability | | | | | | | | |
| MSS | 1.336 | 1.083 | 1.729 | 0.009 | 1.238 | 0.968 | 1.584 | 0.090 |
| MSI |  |  |  |  |  |  |  |  |
| LATS1/2  High vs Low | 1.157 | 0.917 | 1.461 | 0.219 |  |  |  |  |
| CD8  Low vs High | 0.706 | 0.559 | 0.893 | 0.004 | 0.705 | 0.556 | 0.893 | 0.004 |
| FOXP3  High vs Low | 1.110 | 0.878 | 1.402 | 0.384 |  |  |  |  |
| CD163  High vs low | 1.222 | 1.089 | 1.417 | 0.033 | 1.040 | 0.828 | 1.324 | 0.840 |

AJCC: American Joint Committee on Cancer; N0: no lymph node metastasis; N1: lymph node metastasis; M0: no distant metastasis; M1: distant metastasis; MSS: microsatellite stability; MSI: microsatellite instability; HR: hazard ratio; CI: confidence interval

**Table S3. Univariable analysis of LATS1/2, CD8, FOXP3 and CD163 in MSS and MSI gastric cancer**

| Clinicopathological Factors | MSS | | | | MSI | | | |
| --- | --- | --- | --- | --- | --- | --- | --- | --- |
|  | HR | 95%CI | | P-value | HR | 95%CI | | P-value |
| LATS1/2  High vs Low | 1.304 | 1.035 | 1.643 | 0.024 | 0.976 | 0.604 | 1.579 | 0.259 |
| CD8  Low vs High | .  0.767 | 0.609 | 0.967 | 0.019 | 0.760 | 0.472 | 1.224 | 0. 133 |
| FOXP3  High vs Low | 1.320 | 1.047 | 1.665 | 0.025 | 1.097 | 0.670 | 1.797 | 0.713 |
| CD163  High vs Low | 1.215 | 0.931 | 1.588 | 0.152 | 1.617 | 1.000 | 2.614 | 0.184 |

MSS: microsatellite stability; MSI: microsatellite instability; HR: hazard ratio; CI: confidence interval

**Table S4. Relationship between LATS1/2 and FOXP3, CD163 and CD8**

| Clinicopathological factors | LATS1/2 | | |
| --- | --- | --- | --- |
|  | low | high | P-value |
| CD8 |  |  |  |
| low | 118 | 127 | 0.008 |
| high | 146 | 99 |  |
| FOXP3 |  |  |  |
| low | 163 | 114 | 0.012 |
| high | 101 | 112 |  |
| CD163 |  |  |  |
| low | 123 | 110 | 0.356 |
| high | 141 | 116 |  |

| Clinicopathological factors | | | Univariate analysis | | | |
| --- | --- | --- | --- | --- | --- | --- |
|  |  |  | HR | 95%CI | | P-value |
| LATS1/2 | CD8 | |  |  |  |  |
| high | low | | 1.639 | 1.203 | 2.232 | 0.005 |
| Others1 | | |  |  |  |  |
| LATS1/2 | FOXP3 | |  |  |  |  |
| high | high | | 1.530 | 1.171 | 2.000 | 0.003 |
| Others2 | | |  |  |  |  |
| CD8 | FOXP3 | |  |  |  |  |
| Low | high | | 1.570 | 1.181 | 2.087 | 0.004 |
| Others3 | | |  |  |  |  |
| LATS1/2 | CD8 | FOXP3 |  |  |  |  |
| high | low | high | 2.207 | 1.653 | 2.959 | 0.001 |
| Others4 | | |  |  |  |  |

### Table S5. [Survival analysis of different subtypes of advanced gastric cancer](https://static-content.springer.com/esm/art%3A10.1186%2Fs12967-019-1929-9/MediaObjects/12967_2019_1929_MOESM2_ESM.docx)

Others1: LATS1/2^high^CD8^high^, LATS1/2^low^CD8^low^, LATS1/2^low^CD8^high^

Others2: LATS1/2^high^FOXP3^low^, LATS1/2^low^FOXP3^high^, LATS1/2^low^FOXP3^low^

Others3: CD8^high^FOXP3^high^, CD8^low^FOXP3^low^, CD8^high^FOXP3^low^

Others4: LATS1/2^high^CD8^low^FOXP3^low^, LATS1/2^high^CD8^high^FOXP3^high/low^, LATS1/2^low^CD8^high/low^FOXP3^high/low^
